# Aging and Coronavirus: Exploring Complementary Therapies to Avoid Inflammatory Overload

**DOI:** 10.3389/fmed.2020.00354

**Published:** 2020-06-26

**Authors:** Leônidas Oliveira Neto, Vagner Deuel de Oliveira Tavares, Nicole Leite Galvão-Coelho, Felipe Barreto Schuch, Kenio Costa Lima

**Affiliations:** ^1^Department in Arts, Federal University of Rio Grande do Norte, Natal, Brazil; ^2^Laboratory of Hormonal Measurements, Department of Physiology and Behavior, Brazil and National Institute of Science and Technology in Translational Medicine, Federal University of Rio Grande do Norte, Natal, Brazil; ^3^Department of Sports Methods and Techniques, Federal University of Santa Maria, Santa Maria, Brazil; ^4^Department of Odontology, Federal University of Rio Grande do Norte, Natal, Brazil

**Keywords:** lifestyle, nutraceuticals, coronavirus, health, inflammation

## Overview

Acute respiratory distress syndrome (ARDS) is the main cause of death in COVID-19 patients (1, 2). In recent years the relationship between this respiratory syndrome and inflammatory system dysregulation has been discussed (3). Patients with ARDS could present distinct endophenotypes with respect to immune alterations: hyper- or hypo-inflammatory profiles (4, 5).

The identification of inflammatory endophenotypes of ARDS is important, as patients respond differently to clinical and hospital management (3). In patients with a hyper-inflammatory profile, a pro-inflammatory storm is observed in the human body, with elevated rates of biomarkers such as C Reactive Protein (CRP) (2, 6) and cytokines such as interleukins (IL)-6 and tumoral necrosis factor (TNF)-α that are able to develop a systemic inflammatory response. The release of IL-6 and TNF-α into the systemic circulation directly contributes to the increase in systemic inflammation levels and arteriosclerosis processes (7).

People with chronic clinical comorbidities (1) such as hypertension, diabetes (8), and kidney disease (9) have a higher risk of becoming critically ill and dying from COVID-19. For this reason, the older age population has a higher risk of mortality by COVID-19, since they have many of these diseases (10, 11). It is interesting to highlight that both aging and chronic diseases are linked to an increase in levels of systemic inflammation, which could explain a potential common pathway between these factors and COVID-19. Therefore, the acute and strong immune system dysregulation induced by the virus may be linked to ARDS and its complications, such as multiple organ failure, and finally lead to patient death (12), mainly in those with previous inflammatory allostatic overload (13, 14).

In fact, people with COVID-19 present high levels of systemic inflammatory biomarkers (15), and the detection of these forms part of the preliminary guidelines for the diagnosis and treatment of SARS-CoV-2 (12). Accordingly, multiple experimental treatments with immune-suppressing or stimulating drugs have been tested, aiming to reduce the pro-inflammatory cascade and, thus, mortality (16–18).

While the search for effective treatments and vaccines is the top priority, non-pharmacological complementary therapies targeting reductions in baseline inflammatory load, mainly in the oldest population, should receive some attention. During aging, a natural and progressive deterioration in cells and impairment in organ functions occur due to metabolic, immunological, neuroendocrine, or oxidative stress (19). At a molecular level, imbalance between the oxidant/antioxidant pathways (19) could be explained by malfunction in inflammatory/anti-inflammatory homeostatic mechanisms, which result in a chronic low-grade pro-inflammatory state known as *inflammaging* (20).

The inflammatory system is responsible for defending systemic functioning and repairing damages from infections and harmful environmental agents. Aging is a process that all living organisms ages and corresponds to a reduction of defenses to the aggressor agents of living beings, and this we call immunosenescence. This process is gradual and differs between genders (21). At ~40 years of age, the first major reduction in immune functions occurs and occurs in a similar way between men and women. Studies with COVID-19 reveal that it is exactly in this age group that lethality doubles, from 0.2 to 0.4%. Around the early post-60s, we have a new functional immune decline for men, which only occurs in the late 60s for women, which may partly explain the higher mortality of men worldwide (22). Several studies report that, with aging, both the innate and adaptive immune response suffer changes both in their cellular composition and in their function (23, 24). In the case of COVID-19, the innate immune response in the elderly would be activated, and there would be no satisfactory passage of the innate immune response to adaptive, maintaining a chronic activation of the former and preventing the elimination of SARS-CoV2 (23, 25).

In addition to maintaining the chronic immune response, which generates a chronic inflammatory state, there is an important decline in the performance of the adaptive system. Yet, there is a reduction in the recognition of new antigens by adaptive immunity due to the reduction of naive cells and, moreover, a depletion of aging immune cells, which are already very stimulated and do not retain their functions. There are reports that immune cells of adaptive response also undergo changes in their functions and start to act as cells of the innate response (26). During the COVID-19 pandemic, two of the pro-inflammatory proteins were elevated in severe patients (27), yet the inflammatory state may be associated with multiple diseases (25).

In this sense, the consequences are systemic and affect the elderly especially, causing changes in body composition and an imbalance between availability and energy demand that can affect the quality of life and functionality of the elderly (28). In addition, the inflammation overload makes the elderly more susceptible to several other diseases, such as cardiovascular disease, diabetes, osteoporosis, and ostearthrosis (29).

In this context, *lifestyle* and *nutraceutical*s arise as important prophylactic interventions to reduce the burden of baseline inflammation in older adults and consequently improve quality of life, mobility, cognition, mood, and metabolic and immune balances, especially during the pandemic. It is possible that COVID-19 will be a long pandemic, with multiple infection waves (30); therefore, these strategies are especially important since they can be adopted in the long term and under physical social isolation.

The aim of this study is to discuss how diet and *nutraceuticals* and *lifestyle* as complementary therapies could help older adults during the COVID-19 pandemic, reducing *inflammaging*.

## Diet and Nutraceuticals

*Comfort foods* are very palatable foods that are rich in saturated fats and carbohydrates, especially sugar, which can decrease stress and anxiety through activation of the dopaminergic pathways of the reward system (31, 32). In times of lockdown, a rise in the intake of comfort foods is likely, and this behavior tends to strengthen each time the reward system is activated (33). Since *comfort foods* have a high caloric rate, they can lead to weight gain when the energy expenditure is lower than the caloric intake, resulting in obesity, which is recognized as an inflammatory disease (34).

In order to avoid weight gain, which adds load to *inflammaging* through an increase in the synthesis of harmful adipocytokines by white adipose tissue (35), a diet should be prescribed by a specialist. For instance, some diets, such as the Mediterranean diet, the low glycemic index diet, moderate carbohydrate intake, and vegetarian diets, should be adapted to the personal demands and preferences of older adults and prescribed in times of lockdown (36). However, diets with severe restriction should be avoided, as they could lead to impulsive food behaviors (31).

Besides adjustment in the diet, some specific nutrient supplementations can assist in health improvement, such as magnesium, zinc, S-adenosyl methionine, omega-3, and vitamin D, which are important for good maintenance of cognitive and physiological mechanisms (37, 38). Magnesium is fundamental for nervous system function and insulin sensitivity, helping in the prevention or management of Diabetes Mellitus type II, characterized as a chronic and mild inflammatory disease (34, 39). Zinc also contributes to improving insulin sensitivity (40) and body metabolism (39). Vitamin D, or more specifically, 25-hydroxyvitamin D [25 (OH) D], is an anti-inflammatory nutrient (41), and reduces the activation of the renin-angiotensin system, preventing hypertension (42), besides its importance to bone and muscle, an inverse relationship is also observed between its levels and mortality risk in old adults (43). Omega-3 has an important role in cognition and as an anti-inflammatory agent; thus, it seems effective against age-related mood disorder (44, 45).

Recently, 25-hydroxyvitamin D [25(OH)D] has been suggested as a *nutraceutical* alternative to reduce the risk of COVID-19 infection due to improvement in the immune system, whereas vitamin D3 is pointed out as an adjunctive treatment in higher doses (1, 46). In addition, vitamin C could be an alternative to treat respiratory tract infections. Also, one study indicated that administration of ~ 15 g/day of vitamin C for 4 days may decrease mortality in patients with ARDS (47). However, the vitamin C supplementation did not significantly improve organ dysfunction scores or alter biomarkers of inflammation and vascular injury. Thus, controlled trials and large-population studies should be conducted to prove these hypotheses.

Moreover, it is important to highlight that the benefits of both diet and *nutraceutical* interventions are enhanced and the risks reduced when planned for a specific patient, through precision-based approaches that consider nutritional macro/micronutrient deficiencies, levels of inflammatory cytokines, and genomic and microbiome analysis, among other factors (48). This individual analysis is mainly relevant to elderly adults who usually show imbalances in many micro- and macronutrient levels as a result of aging or pharmacological treatments. Although some of these approaches are low-cost, unhappily, they are not always applied. Therefore, their use should be stimulated to has to help reduce the number of deaths around the world, mainly during the pandemic (49).

## Sedentary Behavior and Lifestyle Therapy

Sedentary behaviors such as longer screen time and lower physical energy expenditure can aggravate physical and mental conditions (50), especially in this period of social isolation. Therefore, reducing the time spent in sedentary behavior at home is of great importance for maintaining health during lockdown (51). Furthermore, increasing the time spent engaging in exercise is essential.

Lifestyle therapy consists of adopting a health routine that includes a balanced diet, physical exercise, relaxation and meditation techniques, and good sleep (38, 48).

A robust body of evidence has demonstrated the benefits of these modifications of lifestyle for mental health, mainly for mood symptoms (52–55), indicating that lifestyle therapy is an effective strategy for preventing and treating some mental disorders (56–59), including in old adults (45).

It is natural that with aging, the frequency and intensity of physical activities will decrease (51). However, there are further reasons for encouraging an increase in activity levels, such as for improving cardiorespiratory fitness (60), which in turn reduces mortality risk (61), and poor health (62). Furthermore, reducing sedentary behavior and engaging in exercise may increasing the production of systemic anti-inflammatory cytokines and help to combat inflammation (63, 64) by increasing innate immune function (65) and decreasing the chronic inflammation related to various diseases (66).

Considering the high rate of risk factors being present in older adults as a risk group (67), it is necessary to build tools directed at this group that aim to reduce sedentary behaviors and to keep them active during the COVID-19 pandemic. As well as setting prescribed exercises and encouraging increased levels of daily physical activity, all movements should be stimulated, even simple routine activities such as those related to cleaning the house (68).

With respect to exercises, to reduce sedentary behavior, we recommend the practice of modest exercises that are popularly known as jumping jacks, going up and down stairs, pushups, sit and get up, and balance exercises. These exercises are options that can fit well into the lockdown situation and can be done with home objects such as chairs and benches. However, all exercise should be supervised and prescribed by a trained professional, considering the individual, social, and economic aspects of the subject. However, it is necessary that this orientation occurs using distance-oriented tools, such as internet-based strategies like apps or video calls or mobile telephone messages. Group classes can also improve motivation and social support, which in turn reduces psychological stress levels, helping in homeostatic balance (69).

However, as some elderly adults have impaired motor skills, other alternatives have been used to reduce symptoms of mental disorders and reduction inflammation. For this, approaches with an integrative mind-body focus have been gaining ground in order to prevent or treat diseases such as chronic stress, anxiety, and depression (70), which are known to induce a mildly pro-inflammatory profile (71). These approaches use meditative practices as tools aimed at refining attention and promoting better emotional regulation and self-awareness (72). One of the main components of mindfulness-based activities is the regulation of attention (73). Thus, attentional focus during the exercises proposed in mindfulness programs is directed to the observation of the experience of thoughts, body sensations, and emotions (74, 75). In addition, the practice of relaxation and meditation also has an effect on reducing inflammation (76).

Successful mind-body interventions in older adults have shown improvements in different aspects, such as pain control, sleep quality, attention, global cognition, and working memory (77). Additionally, positive results were recently presented for the reduction of depressive symptoms through internet mindfulness therapy in this population (78). Therefore, applying relaxation and meditation therapies is urgent, as these can improve mental and physical health in older people who are in isolation, following the guidelines of the WHO.

## Conclusion

Social physical isolation due to COVID-19 can bring serious risks to health if older adults continue with, or assume, a *non-healthy lifestyle*, which includes a lack of physical activity and a diet low in nutrients and rich in *comfort food*s. Therefore, strategies should be encouraged to promote and raise awareness among the older population about the application of *lifestyle* and *nutraceutical* tools. These interventions have great potential for insertion in public policies in different contexts due to their low cost, effectiveness, and simplicity. We are aware that it can be difficult to apply all of these suggestions, mainly in elderly adults, but every step is important and better than none. Therefore, a healthy lifestyle should be encouraged as an intervention to prevent frailty among older people, and a multi-professional care system should act in this time of COVID-19 to reduce risks and avoid damage related to inflammation overload in older adults.

## Author Contributions

LN: conceptualization, project administration, and writing—original draft preparation. VT, NG-C, and FS: reviewing and editing. KL: conceptualization, project administration, and writing—original draft preparation. All authors contributed to the article and approved the submitted version.

## Conflict of Interest

The authors declare that the research was conducted in the absence of any commercial or financial relationships that could be construed as a potential conflict of interest.
